# Supplementary material for: Demonstration of Ru as the 4th ferromagnetic element at room temperature
Source: Nat Commun. 2018 May 25;9:2058. doi: 10.1038/s41467-018-04512-1 (PMC5970227; doi:10.1038/s41467-018-04512-1)
Supplement: Supplementary file 1 — Supplementary Information [file 41467_2018_4512_MOESM1_ESM.pdf]

Demonstration of Ru as the 4th ferromagnetic element at room temperature

Supplementary materials

Quarterman *et al*

Demonstration of Ru as the 4th ferromagnetic element at room temperature

Supplementary materials

Authors: Paige E. Quarterman[1], Congli Sun[2], Javier Garcia-Barriocanal[3], Mahendra DC[4], Yang Lv[1], Sasikanth Manipatruni[5], Dmitri E. Nikonov[5], Ian A. Young[5], Paul M. Voyles[2], and Jian-Ping Wang[1]*

Affiliations:

[1]Department of Electrical and Computer Engineering, University of Minnesota, MN 55455, USA

[2]Department of Materials Science and Engineering, University of Wisconsin, WI 53706, USA

[3]Characterization Facility, University of Minnesota, MN 55455, USA
[4] School of Physics and Astronomy, University of Minnesota, MN 55455
[5] Components Research, Intel Corp., Hillsboro, OR 97124, USA

*Email: jpwang@umn.edu, Tel. 612-625-9509

Supplementary Note 1. Additional thin film characterization

The surface profile, as measured by AFM (Supplemental Fig. 1) shows each sample is smooth, with all having a roughness (RMS) less than 3 Å, which in addition, confirms the validity of the results obtained from XRR fitting. Additional cross section STEM images of 2.5 and 12 nm Ru samples were collected (Supplemental Fig. 2). The 2.5 nm Ru shows consistent BCT Ru phase. In the 12 nm Ru sample there is a distinct BCT Ru region, but also a region that cannot be definitively identified by high resolution STEM because it contains overlapping BCT and HCP phases. Regions with no distinct phase, such as in the 12 nm sample, are also found in the 2.5 and 6 nm Ru films, but the frequency of these regions increases with thickness. This shift to a greater amount of HCP grains with increased thickness is further supported by XRD scans (Fig 1c).

Supplementary Fig. 1: Surface profile of Ru

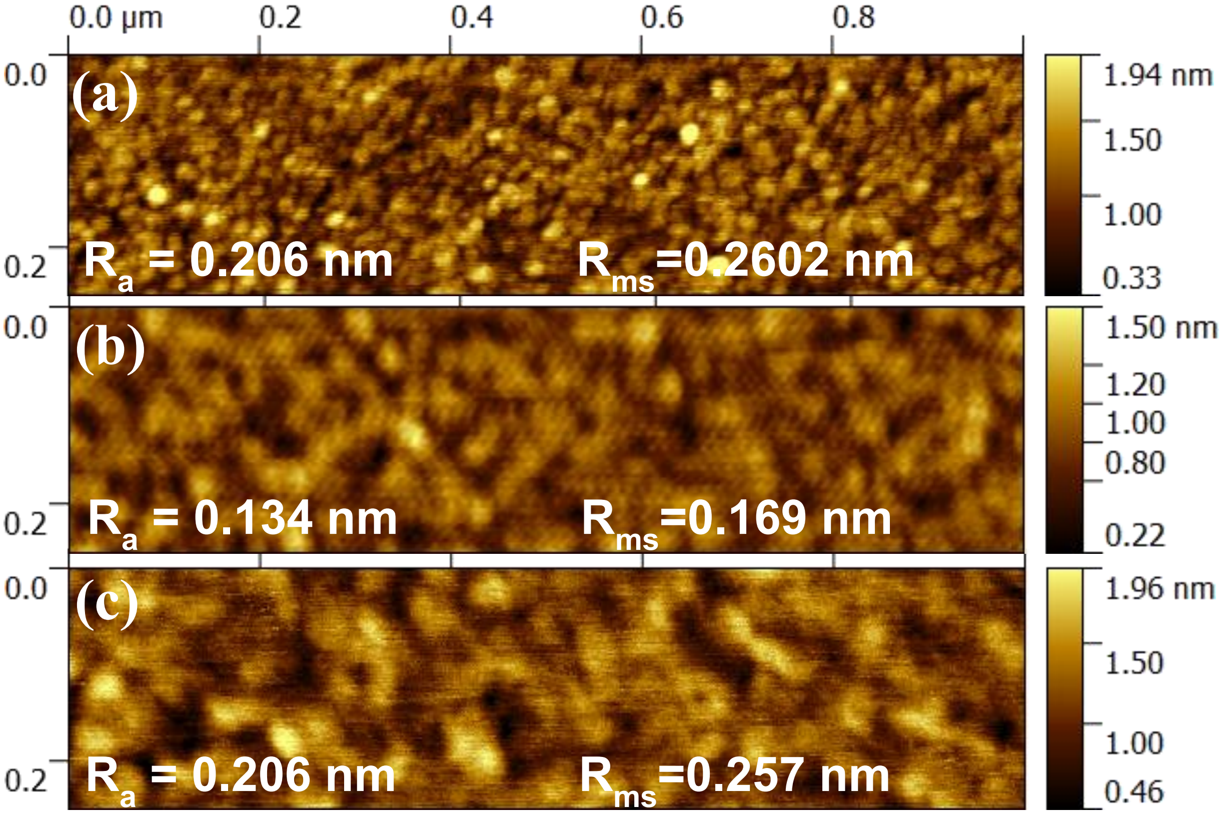

AFM images for (a) 2.5, (b) 6 and (c) 12 nm Ru films show a smooth Ru surface

Supplementary Fig. 2: Additional cross section STEM

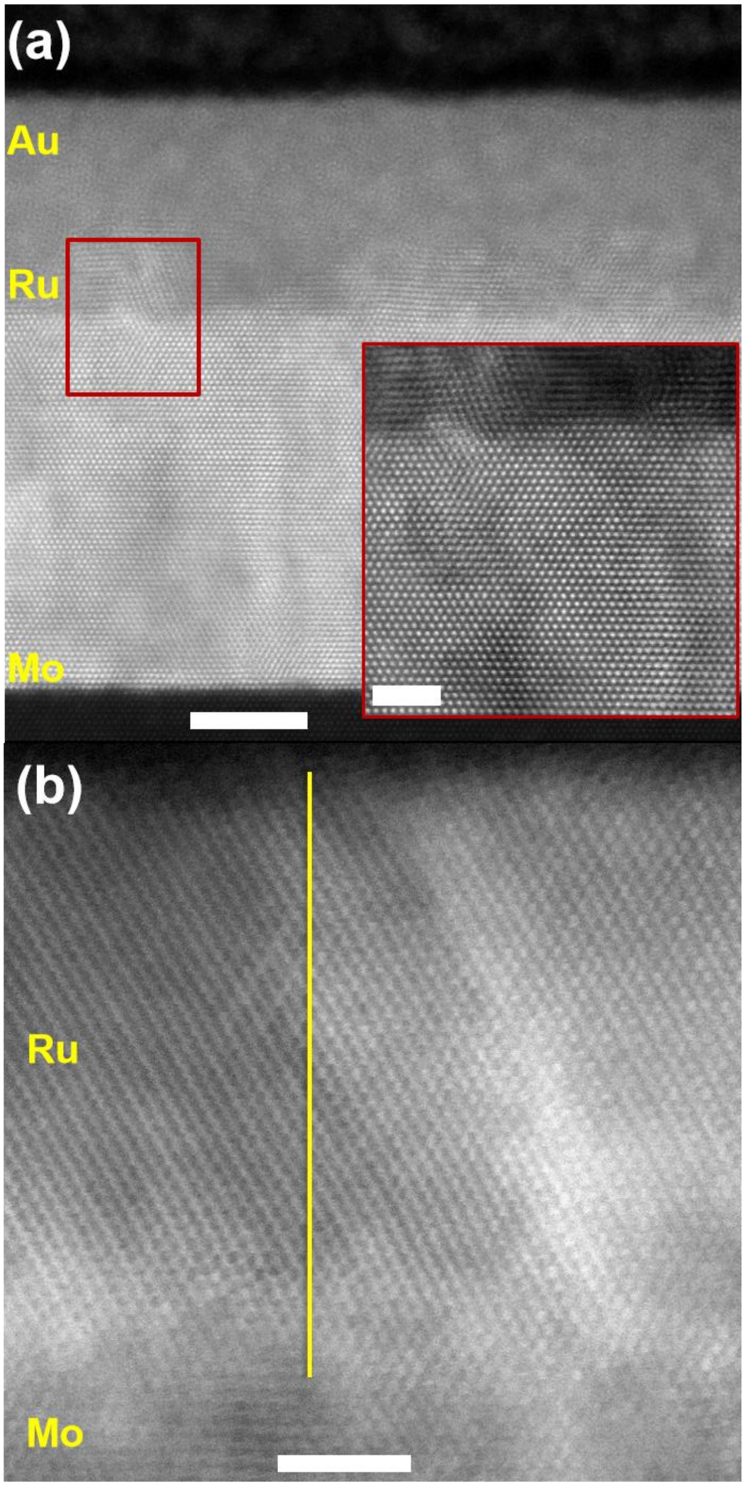

Cross section STEM images along the [001] zone axis of Al_2O_3 for (a) 2.5 nm with the inset showing a zoom in of the boxed region (scale bar is 5 nm and inset is 2 nm) (b) 12 nm Ru (scale bar is 2 nm). In the 12 nm Ru sample, two regions with differing crystallographic structure can be seen, as denoted by regions 1 and 2. Region 2 shows BCT structure akin to that shown in Fig. 2, however, region 1 is not clearly a tetragonal or hexagonal structure, but

Supplementary Note 2. Magnetic control measurements

It has been confirmed that the ferromagnetic properties observed were not due to contamination from the VSM sample holder or impurities in the sputtering target. This was accomplished by measuring the moment vs field curve for the holder used for each Ru after acquiring the sample hysteresis curve, a typical observed curve for the holder is shown in Fig. S3a, which clearly shows only diamagnetism from the sample holder. Contamination in the film as a source of ferromagnetism was ruled out by measuring moment vs. field for a 2.5 nm Ru film, with identical Mo seed layer and substrate, but with no texture, as confirmed in Fig. 1c. The moment vs. field curve (Supplemental Fig. 3b) confirms the observed ferromagnetism is indeed from the metastable Ru film, and not magnetic contamination, since contamination would show ferromagnetic behavior regardless of the texture quality. A total of five 2.5 nm, five 6 nm, and 12 nm Ru samples were grown. In addition, five control samples were grown that should contain no ferromagnetism and are used to rule out contamination as the source of observed ferromagnetism (e.g. no texturing in thin films, or no Ru layer). Supplemental Table 1 summarizes the total samples grown, and a combined total of 11 out of 12 samples for 2.5, 6 and 12 nm samples show ferromagnetic behavior similar to results shown in Fig. 4. In total, 0 out of 5 control samples (those with no texture, and (110) textured Mo with no Ru layer) displayed ferromagnetism. The sample that does not clearly show ferromagnetism was sputtered on a 2-inch Al_2O_3 wafer, whereas all other samples in this work were grown on 5 x 5 mm^2 Al_2O_3 squares. Each sample was measured multiple times at both room temperature and 10 K, the total number of ferromagnetic M vs. H hysteresis loops for each sample is also noted in Supplemental Table 1.

Supplementary Fig. 3: Magnetic control measurements

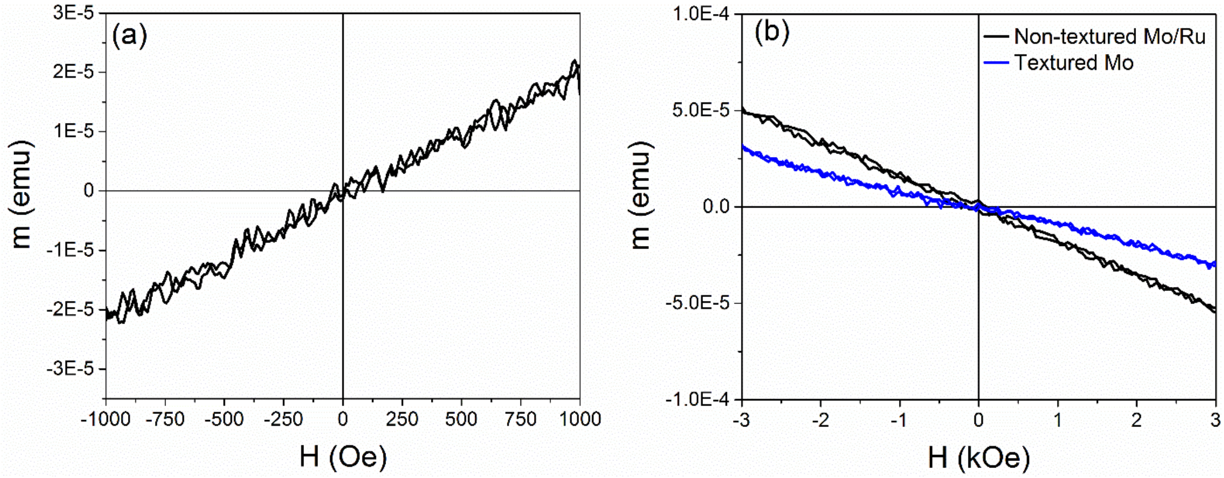

Moment vs field for (a) VSM sample rod, r (b) non-textured Ru (black) and textured (110) Mo with no Ru (blue) control samples. Both curves show no ferromagnetism, and correspond to the 2.5 nm sample. The 6 and 12 nm sample holder show similar non-ferromagnetic behavior.

Supplementary Table 1: Summary of Ru samples

**Sample** Sub\Mo(20)\Ru(X)	**2.5 nm**	**6 nm**	**12 nm**	**Control**	**Total FM samples**
**# Made**	5	5	2	5	12
**# FM**	4	5	2	0	11
**# FM M vs. H**	30	21	4	5	55

The total number of samples fabricated (top row) are displayed in the '# Made' row and the number of these samples that display ferromagnetic (FM) behavior, such as in Fig. 4, are counted in the #FM row. Finally, the total number of ferromagnetic hysteresis loops measured across multiple measurements of the combined samples of the same thickness is counted in the '#FM M vs. H' row.

Supplementary Note 3. Differences in M-H and R_{Hall}-H

The most obvious difference between the **M-H** and **R_{Hall}-H** measurements is the resulting M_s calculated. As mentioned in the main text, this is due to the inherit difference in calculating

M_s between the two measurement methods. In the case of **M**-**H**, M_s is calculated by dividing the measured magnetic moment by the appropriate magnetic volume. This calculation relies on the assumption that the Ru thin film is uniformly magnetic, which from XRD and STEM results, we know to be an invalid assumption. Due to this error in the volume, it is not surprising that a smaller than predicted M_s has been calculated from the **M**-**H** measurements. Determination of Ms from $\mathbf{R}_{Hall}$-**H** measurements instead relies on the assumption of the shape anisotropy for a thin film as that of an infinite plane, which is a valid assumption given the low surface roughness and uniformity of the Ru thin films observed.

Additionally, it should not be expected for the **M**-**H** and $\mathbf{R}_{Hall}$-**H** measurements to have the same shape. In ferromagnets, $\mathbf{R}_{Hall} = \mathbf{R}_O + \mathbf{R}_{AHE}$, and so in the switching field region, R_{AHE} is expected to depend linearly on the field since $\mathbf{R}_{AHE}$ is directly dependent on M_z, which is proportional to H_z. Thus, $\mathbf{R}_{Hall}$ will linearly increase with the field until the magnetization is fully saturated; which in the case of a thin film with no easy axis, and a field applied perpendicular to the surface, will occur at $\mathbf{H} = 4\pi M_s$. The switching region of the **M**-**H** curve, however, is not linearly dependent on the field since the field is not being applied along a hard anisotropy axis. Similar methods have been used to demonstrate trace levels of ferromagnetism by Philip et al[1] and Matsukura et al[2].

Finally, it should be noted that in the saturated region of the $\mathbf{R}_{Hall}$-**H** measurement a linear background with negative slope has been subtracted. This negative slope is due to the ordinary Hall effect of Mo and Ru layers, which are electron carrier dominant since the layers are metallic; this same negative linear dependence on field can be seen in the non-textured $\mathbf{R}_{Hall}$-**H** control measurement.

Supplemental references

1. Philip, J. *et al.* Carrier-controlled ferromagnetism in transparent oxide semiconductors. *Nat. Mater*. **5,** 298–304 (2006)

2. Matsukura, F., Ohno, H., Shen, A. & Sugawara, Y. Transport properties and origin of ferromagnetism in (Ga,Mn)As. *Phys. Rev. B.* **57,** R2037–R2040 (1998)
